# The role of intestinal microbiota and its metabolites in the occurrence and intervention of obesity

**DOI:** 10.3389/fmicb.2025.1559178

**Published:** 2025-05-02

**Authors:** Yang Gao, Wenhui Liu, Xue Ma, Min Xue, Yingying Li, Shanshan Bi, Huan Leng

**Affiliations:** ^1^College of Life Science, Baicheng Normal University, Baicheng, China; ^2^Xiamen Jinfen Agricultural Research Institute, Xiamen, China; ^3^Terra Research and Teaching Centre, Microbial Processes and Interactions (MiPI), Gembloux Agro-Bio Tech, University of Liège, Gembloux, Belgium; ^4^Key Laboratory of Development and Application of Rural Renewable Energy, Biogas Institute of Ministry of Agriculture and Rural Affairs, Chengdu, China

**Keywords:** obesity, metabolic diseases, gut microbiota, metabolic disorders, gut microbial metabolites

## Abstract

Obesity is a growing global health challenge influenced by genetic and environmental factors. With economic development, obesity rates continue to rise globally. Recent research highlights the critical role of gut microbiota in obesity pathogenesis, particularly through microbial metabolites affecting metabolism and energy homeostasis. Recent studies indicate that gut microbiota significantly influences obesity development through metabolic interactions, including the regulation of glucose and lipid metabolism. Gut microbiota is involved in the metabolism of various nutrients such as glucose and lipids, and the change of gut microbiota leads to insulin resistance, adipose tissue accumulation and metabolic disorders, thus mediating the occurrence and development of metabolic diseases. For example, beneficial bacteria such as *Bifidobacterium* and *Lactobacillus*, which are in high abundance in animal intestinal tract, can prevent obesity by enhancing the intestinal barrier, improving insulin sensitivity and improving metabolic disorders. Many of these effects are mediated by metabolites produced by intestinal microbiota, such as short-chain fatty acids, which play an important role in preventing and treating obesity. In this research review, we used the PubMed database and Google Scholar and web of science to search for relevant literature within recent 20 years on the topic of microbes and microbial metabolites in relation to obesity. Key words used were obesity, metabolic diseases, gut microbiota, metabolic disorders, gut microbial metabolites to search for references. The purpose of this review article was to describe the current knowledge on the impact of gut microbiota and their metabolites on obesity, in order to provide a more comprehensive understanding of the relationship between gut microbiota and obesity for future clinical intervention and prevention of obesity.

## 1 Introduction

There are approximately 100 trillion bacteria in the human intestine, which make up the intestinal microbiota. The composition of the intestinal microbes is affected by pH value, oxygen and nutrients (Suau et al., 1999). The intestinal microbiota plays a key role in many physiological and pathological processes in humans (Becattini et al., 2016). Disturbances in the intestinal flora are closely related to the occurrence of metabolic diseases (Sittipo et al., 2018). There is increasing evidence that the intestinal microbiota regulates the body responses by improving the intestinal micro-environment and plays a key role in physiological homeostasis (Vallianou et al., 2021).

The composition of the intestinal microbe is mainly affected by environment and genetic factors (Rothschild et al., 2018). When genetic factors or environment change especially diet change, the intestinal microbial diversity will also change, which is called intestinal microbial imbalance (Vallianou et al., 2021). Conversely, intestinal microbial imbalance may also lead to metabolic disorders in the host, further causing the occurrence of metabolic diseases such as obesity, nonalcoholic fatty liver disease (NAFLD) and diabetes (Biedermann and Rogler, 2015; Hoozemans et al., 2021). A study has shown that animals of the same species with different body weights have significant differences in the composition of their intestinal microbiota (Saad et al., 2016). Intestinal microbial fermentation degrades carbohydrates in the intestine and produces short-chain fatty acids (SCFAs), such as acetate, propionate and butyrate (Cummings et al., 1987). The intestinal microbiota can affect lipid metabolism through the metabolites of short-chain fatty acids. Studies have shown that SCFAs are associated with the prevention and treatment of obesity-related insulin resistance (De Vadder et al., 2014; Mollica et al., 2017). The reduction of SCFAs may play an important role in reducing insulin sensitivity and the occurrence of obesity. In addition, SCFAs exert beneficial effects on body weight, inflammation, insulin sensitivity and glucose homeostasis (Canfora et al., 2017; Bouter et al., 2018). Apart from short-chain fatty acids, lipopolysaccharide, peptidoglycan, and trimethylamine are also important gut microbial metabolites, which play important role in intestinal homeostasis.

In this article, we mainly talk about three parts, the microbes preventing obesity, the microbes promoting obesity and the microbial metabolites with their effects. This review aims to discuss the effects of beneficial intestinal microbes such as *Lactobacillus*, *Bifidobacterium* and harmful bacteria such as *Enterobacter clocae,* as well as microbial metabolites on obesity in order to provide scientific reference for the prevention and treatment of obesity and metabolic diseases.

## 2 Gut microbiota, microbial metabolites and obesity

Currently, with the increasing incidence of obesity, it has become a major health problem from all over the world, affecting the occurrence of many chronic diseases, such as NAFLD, hyperlipidemia and diabetes (Scherrer et al., 2018; Cuthbertson et al., 2021). A study has shown that the intestinal flora plays a direct role in the development of obesity by regulating host genes involved in energy storage and consumption, thereby changing energy and fat storage (Tsukumo et al., 2015). Under normal physiological conditions, lipogenesis and lipolysis maintain a dynamic balance, excessive high-fat diet is the main cause of this imbalance (Wang et al., 2021), which may lead to lipid metabolism disorders. Activated Protein Kinase (AMPK) is an intracellular energy sensor involved in lipid metabolism. A high-fat diet can inactivate AMPK by regulating target genes, leading to excessive accumulation of fat in the body. In recent years, more and more studies have found that probiotics can improve lipid metabolism disorders induced by high-fat diet through the AMPK signaling pathway. GPR43 is an important member of G protein-coupled receptors (GPRs) involved in lipid metabolism in adipocytes. In an experiment on mice fed a high fat diet (HFD), GPR43 levels increased significantly after drug treatment. Its mechanism may be to regulate AMPK activity through the Ca^2+^/CAMKK pathway. BAA6 activates peroxisome proliferator-activated receptor *α* (PPARα) by targeting the acetic acid produced by the GPR43/AMPK signaling pathway, thereby promoting fatty acid oxidation (Ray et al., 2018). At the same time, a study has found that probiotics can significantly inhibit the activity of acetyl-CoA carboxylase (ACC) by activating the AMPK signaling pathway, thereby directly inhibiting lipid synthesis (Rahman et al., 2021). In addition, a high-fat diet can lead to excessive secretion of leptin by adipocytes. High levels of leptin can directly inhibit the activation of AMPK signaling, resulting in lipid metabolism disorders (Lindholm et al., 2013). A study has shown that probiotics can improve the development of obesity and metabolic diseases by inhibiting the secretion of leptin (Jeung et al., 2018). Probiotics can not only break down fats, but also promote digestion and metabolic functions by regulating intestinal microorganisms.

### 2.1 Microbes preventing obesity

#### 2.1.1 Lactobacillus

In recent years, *Lactobacillus* has been a research hotspot in the *Firmicutes*, it can be used to treat obesity and improve intestinal microbial diversity (Song et al., 2021). In a study on preventing obesity by regulating the gut microbiota and protecting intestinal health, the proportion of *Firmicutes* in mice increased from 65 to 78%, while the proportion of *Bacteroidetes* decreased from 26 to 11% during the occurrence of obesity (Han et al., 2021). The ratio of *Firmicutes* to *Bacteroidetes* (F/B) in the mouse intestine is also an important factor affecting obesity (Ley et al., 2006). When the rate of F/B increases, the expression of polysaccharide digestive enzymes in the intestine increases, thereby improving the efficiency of obtaining energy from food and making obesity more likely to occur (Turnbaugh et al., 2006). A study has shown that the F/B ratio was increased in diabetic obese mice compared with normal mice. Among probiotics, *Lactobacillus* and *Bifidobacterium* are relevant to the treatment of metabolic syndrome. It exhibits significant anti-obesity effects in rodents and humans, for example regulating body weight, improving blood glucose and lipid metabolism, reducing insulin resistance (Gibson et al., 2017). *Lactobacillus rhamnosus CGMCC1.3724* combined with a low-calorie diet resulted in significant weight loss in obese women compared with obese men (Sanchez et al., 2017). A study has shown that *Lactobacillus reuteri* intervention can significantly inhibit the weight gain of mice, while mice supplemented with *Lactobacillus reuteri L6798* have a significant increase in body weight (Fåk and Bäckhed, 2012). Other study has found that (Kang et al., 2022) *Lactobacillus sachusetts* can reduce the levels of total cholesterol (TC), triglyceride (TG) and lipopolysaccharide (LPS) in the blood, while inhibiting the activation of nuclear factor kappa-B (NF-κB) and the expression of tumor necrosis factor *α* (TNF-α) induced by a high-fat diet. In obese mice, *Lactobacillus sarcopeniae* reduces obesity by regulating intestinal microiota, inducing AMPK activation, and increasing the expression of peroxisome proliferator-activated receptor-*γ* coactivator-1α (PGC-1α). The study by Kang et al. (2022) showed that *Lactobacillus acidophilus* down-regulated the expression of proinflammatory cytokines such as TNF-α, interleukin-1β (IL-1β) and interferon-γ (IFN-γ) in mice fed a high-fat diet, and up-regulated the expression of key transcription factors involved in lipid metabolism such as PPARα, PPAR-γ and C/EBP-α. By inhibiting the expression of NF-κB, thereby improving insulin sensitivity and restoring the ratio of *Firmicutes* to *Bacteroidetes* in the intestine, *Lactobacillus* has now become a candidate probiotic for improving obesity and related diseases (such as hyperlipidemia and non-alcoholic fatty liver disease) (Wang et al., 2023; Lau et al., 2024).

#### 2.1.2 Akkermansia muciniphila

Studies have shown that the diversity of intestinal microbiota in obese mice is significantly lower than that in normal mice (Li et al., 2022). In addition to the *Firmicutes* and *Bacteroidetes*, *Akkermansia muciniphila* has also received extensive attention and research, it has been reported to have closely links with human obesity (Lee et al., 2019). In recent years, more and more studies have shown that the relative abundance of *Akkermansia muciniphila* has a significant negative correlation with obesity-related indicators such as body mass index (BMI) and waistline (Dao et al., 2016; De La Cuesta-Zuluaga et al., 2017; Journey et al., 2020). Higher levels of *Akkermansia muciniphila* correlate with leanness by enhancing metabolic rate and restoring intestinal barrier function (Plovier et al., 2017). In a recent study of overweight women with PCOS, the abundance of *Akkermansia muciniphila* in the intestines of women who ate a healthy diet and had a lower BMI (Łagowska et al., 2022). One study showed that mice treated with anthocyanins not only had healthier intestines, but also contained a higher proportion of *Akkermansia muciniphila* (Li et al., 2022) which was able to reduce inflammation and obesity by reducing lipid deposition and inhibiting the proportion of macrophages (Xie et al., 2021). In addition, Marvasti et al. (2020) first reported that the relative abundance of *Akkermansia muciniphila* was significantly reduced in obese people in Iran. Therefore, *Akkermansia muciniphila* is beneficial to intervene and treat human obesity as well as metabolic diseases.

#### 2.1.3 Bifidobacterium

Currently, a large number of studies have shown that *Bifidobacteria* play a beneficial role in improving obesity-related symptoms (Gibson and Roberfroid, 1995; Kalliomäki et al., 2008). The increased abundance of *Bifidobacterium* in the intestine can increase the content of SCFAs, reduce the content of LPS and improve the intestinal barrier function. Compared with normal person, the abundance of *Bifidobacterium* in the intestine of obese people is significantly reduced (Singh et al., 2017). Oral *Bifidobacterium* to mice can reduce their weight gain (Schroeder et al., 2018; Zou et al., 2018) and prevent metabolic disorders induced by a high-fat diet (Mischke et al., 2018). Supplementation with *Bifidobacteria* can reduce the levels of TC, TG and glucose, at the same time effectively reduced the body weight and fat deposition. This effect was mainly due to the metabolites of *Bifidobacteria* regulating low-density lipoprotein cholesterol (LDL-C), thereby improving the abnormal fat metabolism in obese mice (Chen M. et al., 2021). In another study, supplementation with *Bifidobacterium* improved antioxidant capacity in overweight women, possibly due to the interaction of short-chain fatty acids with G protein-coupled receptors, affecting adipocyte sensitivity to insulin, thereby regulating energy metabolism (Gomes et al., 2017). The use of *Bifidobacterium* improved dyslipidemia caused by obesity, possibly by inhibiting the expression of intestinal adipokines during the fasting period, thereby further affecting the production of short-chain fatty acids (Gomes et al., 2017). In summary, *Bifidobacterium* has important research value in the prevention and treatment of obesity.

### 2.2 Microbes promoting obesity 2.2.1 *Enterobacter clocae*

In addition to the above-mentioned beneficial bacteria that can prevent and alleviate obesity, there are also some intestinal flora that are considered to be potential bacteria that cause obesity. For example, *Enterobacter clocae* is a Gram-negative bacterium isolated from the intestinal microbiota of obese people. When oral gavage it into germ-free mice, resulting in obesity and insulin resistance (Fei and Zhao, 2013). Another study on 123 obese people who used dietary intervention to control intestinal microbiota showed that in the process of weight loss, the relative abundance of *Enterobacteriaceae* and *Desulfovibrionaceae* in the intestine decreased. At the same time, the relative abundance of *Escherichia* and *Shigella* was positively correlated with systolic blood pressure, *Klebsiella* was positively correlated with fasting blood glucose (FBG), *Citrobacter* was positively correlated with body weight, BMI and IL-1β (Xiao et al., 2014). The study found that acute lung infection induced by intranasal drip of *Escherichia coli* can aggravate lipid metabolism disorders in obese mice. Meanwhile, serum free fatty acids (FFA), TG and very low density lipoprotein (VLDL) were significantly increased after infection, while TC, LDL-C and high density lipoprotein cholesterol (HDL-C) levels showed a downward trend after infection, among which HDL-C decreased the most (Adeghate, 2004). As a lipoprotein, HDL-C not only promotes the transport of cholesterol, but is also very important in the process of anti-infection. Its reduction may aggravate the disorder of blood lipid metabolism in obese mice. In addition, intestinal microbial diversity imbalance is positively correlated with endotoxin content. When the abundance of *Escherichia coli* increases and the dominant bacteria decreases significantly, the intestinal mucosal structure is destroyed and the level of enteric endotoxin gradually increases. Serum endotoxin can enter the liver through the portal vein, inducing hepatocytes to produce inflammatory cytokines such as tumor necrosis factor and interleukin-1, thereby promoting the development of non-alcoholic fatty liver disease (Cui et al., 2019). Therefore, reducing the relative abundance of *Escherichia coli* is also an effective way to intervene and treat obesity and metabolic diseases. The relationships between gut microbiota and obesity-related phenotypes summarized in this article are shown in Table 1.

**Table 1:** The relationships between gut microbiota and obesity phenotypes.

Phylum	Family	Genus	Phenotypic changes	References
*Verrucomicrobia*	*Akkermansiaceae*	*Akkermansia muciniphila*	BMI↓, LPS↓	Journey et al. (2020), Dao et al. (2016), and Xie et al. (2021)
*Actinobacteri*	*Bifidobacteriaceae*	*Bifidobacterium*	SCFAs↑, IL-4↑, HDL-C↑, LPS↓	Singh et al. (2017), Zou et al. (2018), Mischke et al. (2018), and Chen M. et al. (2021)
			TC↓, TG↓, IR↓, IL-6↓, MCP-1↓	
*Firmicutes*	*Erysipelotrichaceae*	*Allobaculum*	TC↓, LPS↓, TG↓, SCFAs↑	Han et al. (2021), Simon et al. (2015), Han et al. (2021), Han et al. (2021), Liang et al. (2021), and Xiao et al. (2014)
	*Lactobacillaceae*	*Lactobacillus*		
			GLP-1↑, GLP-2↑	
		*Turicibacter*	LPS↑	
	*Erysipelotrichaceae*	*Lachnoclostridium*		
			LPS↑	
		*norank_f__Muribaculaceae*	SCFAs↑, TC↓, LPS↓	
		*Klebsiella*		
			FBG↑, HbA1c↑, L/M↑	
	*Lachnospiraceae*	*Citrobacter*		
			BW↑, BMI↑, IL-1*β*↑	
	*Muribaculaceae*			
	*Enterobacteriaceae*			

*Bacteroidetes*	*Bacteroidaceae*	*Bacteroides*	HDL-C↑, SCFAs↑, BWG↓	Han et al. (2021) and Liang et al. (2021)
			BFR↓, TC↓, TG↓, LDL-C↓	
			LPS↓, TNF-*α*↓, IL-6↓	

### 2.3 Microbial metabolites and their effects

#### 2.3.1 Short-chain fatty acids (SCFAs)

Short-chain fatty acids (SCFAs), are metabolites of intestinal microbiota, particularly acetate, propionate, and butyrate, regulate metabolic homeostasis by modulating gut hormone secretion, energy expenditure, and insulin sensitivity. Their role in obesity prevention and treatment is an emerging area of interest in metabolic research. More than 95% of all SCFAs and are usually present in the intestine at a concentration ratio of 3:1:1 (Sun et al., 2017; Faits et al., 2020). They are mainly produced by the breakdown of carbohydrates such as resistant starch (Feng et al., 2018) and can be converted into each other in the body. Among them, acetic acid and propionic acid are usually transported to the liver to participate in energy metabolism. Acetate can be used for the synthesis of cholesterol, fatty acids and glutamine, propionate is an important substrate for gluconeogenesis and can inhibit the synthesis of fat (Kimura et al., 2019; Blaak et al., 2020). SCFAs play an important role in maintaining the homeostasis of the internal environment. They can maintain the homeostasis of the microbiota and promote digestion and absorption. They can also affect body weight by regulating fat synthesis, increasing insulin sensitivity, improving glucose tolerance and thus affecting lipid metabolism (Blaak et al., 2020). The experimental results of Den Besten et al. (2015) showed that SCFAs activate carnitine palmitoyl transferase 1 (CPT1) to participate in the supply of fatty acid substrates, among which the down-regulation of PPARγ activates the UCP2-AMPK-ACC metabolic pathway, converting fat into fatty acid oxidation. Therefore, short-chain fatty acids play a key role in the pathophysiology of obesity. In addition, SCFAs can reduce intestinal barrier dysfunction and metabolic abnormalities induced by a high-fat diet. A study has reported (Matheus et al., 2017) that butyrate alleviates high-fat diet-induced metabolic disorders in mice by inhibiting insulin resistance, which is related to the secretory function of pancreatic B cells and the intestinal barrier mediated by tight junction proteins.

SCFAs can regulate fat production, improve insulin resistance and reduce free fatty acids in overweight people (Zhu et al., 2020), the mechanism is related to the increased secretion of gastrointestinal hormones. SCFAs mainly regulate energy metabolism by modulating the GPRs pathway, activating the MAPK signal transduction pathway of intestinal epithelial cells, and promoting the secretion of hormones such as 5-hydroxytryptamine (5-HT) and gastroinhibitory peptide (GIP) by L cells in the intestine, thereby increasing satiety, reducing gastric emptying and reducing body weight (Aktar et al., 2020). Butyric acid in SCFAs can be converted to acetyl-CoA in cells and oxidized in the circulation to produce ATP, which can activate the expression of regulatory gluconeogenic genes; Propionate binds to neuronal GPRs, resulting in a decrease in cAMP levels, thereby regulating glucose and lipid metabolism in the body (Morrison and Preston, 2016); Acetate may be converted to acetyl-CoA and participate in the tricarboxylic acid cycle in various peripheral tissues. It may also affect metabolism through increased oxidative capacity via 5′AMP-activated AMPK phosphorylation effects (Yamashita et al., 2009; Maruta et al., 2016). As one of the important signaling molecules regulating intestinal mucosal immunity, SCFAs can regulate PPARγ/GPR-mediated adipogenesis by inhibiting histone deacetylase activity, increasing the activity of triglyceride-degrading lipase and sensitive lipase (Blaak et al., 2020). SCFAs can regulate extracellular specific G protein-coupled receptors, mainly intervening in the free fatty acid receptors GPR41, GPR43 and hydroxycarboxylic acid receptor 2 (HCAR2) present in intestinal endocrine cells, enteric neurons and intestinal epithelial cells. GPR43 is highly expressed in white adipose tissue, neutrophils and pancreatic B cells (Lu et al., 2016). Intestinal microbial diversity imbalance often leads to an imbalance in SCFAs levels, thereby affecting metabolism and causing obesity.

#### 2.3.2 Lipopolysaccharide (LPS)

LPS, which is an important molecule in the cell wall of Gram-negative bacteria, is the main metabolite secreted after the lysis of intestinal microbes. There is approximately 1 g LPS in human intestine (Erridge et al., 2007). In healthy individuals, the intestinal epithelium is an effective barrier against LPS, but in disease states, impaired intestinal epithelial function may cause the translocation of LPS. However, low concentrations of LPS can be detected in the blood of healthy animals and humans, indicating that small amounts of LPS continuously cross the intestinal epithelial barrier to the blood (Laugerette et al., 2011). Studies have shown that a high-fat diet for 4 weeks increased the plasma LPS concentration of mice by two to three times and increased the proportion of LPS containing microbiota in the intestine. Long-term infusion of LPS can induce weight gain in mice, so obesity is related to the increase of LPS in plasma (Cani et al., 2007).

LPS contains lipid A, which can pass the intestinal mucosa through tight junctions. It has no toxic effects, but as a nonspecific immunogen, it interacts with the host after entering the micro-circulation. The secreted bio-active molecules can cause fever and inflammatory response in the body. This inflammatory response interferes with the metabolism of glucose and lipids, promotes the production and storage of adipose tissue, leads to metabolic diseases such as insulin resistance (Yaribeygi et al., 2019). In turn, LPS forms a complex with lipopolysaccharide binding protein, acts on Toll-like receptor 4 (TLR4) on the surface of intestinal immune cells, activates the Myd88/NF-κB signaling pathway, thus affects changes in the structure of the intestinal flora (Larraufie et al., 2018). TLR4 is the main mediator of inflammation and immune response, it is widely distributed on the membrane surface of various immune cells, especially monocytes and macrophages. After LPS binds to TLR4 on macrophages and adipose tissue, it activates the gene expression of pro-inflammatory proteins, such as NF-κB and activator protein 1 (AP-1), through the activation of the MyD88 signaling pathway. It activates a series of inflammatory and immune responses, which in turn affects the normal lipid metabolism process and leads to the occurrence of lipid metabolism-related diseases (Lancaster et al., 2018).

The intestinal mucosa plays an important role in nutrient absorption, maintaining intestinal barrier function, and preventing bacterial translocation (Yuan et al., 2018). Studies have shown that a high-fat diet can change the composition of intestinal microorganisms, damage the intestinal mucosa, alter intestinal permeability, and mediate the occurrence of obesity and insulin resistance (Tomas et al., 2016). Obesity is considered a chronic inflammatory response, LPS can stimulate fat deposition and exacerbate inflammation by activating receptors in adipocytes, such as TLR4. During obesity, intestinal permeability increases, which is manifested by increased levels of inflammatory factors in plasma as well as LPS. LPS is an inducer of inflammation, which activates the NF-κB signaling pathway, leading to chronic inflammation and further increasing fat accumulation.

#### 2.3.3 Bile acid (BA)

BA is an important component of bile. It is synthesized by cholesterol oxidation in the liver via cytochrome P450, it is mainly involved in the absorption of bile and fat-soluble vitamins in the intestine (Chiang, 2002). The primary bile acids produced by the direct decomposition of cholesterol mainly combine with glycine in the human liver, increasing the solubility of bile to play a detoxification role. Primary bile acids in the intestine are synthesized into secondary bile acids under the action of the microbes and metabolized in the intestine. The composition of the bile acid profile is closely related to the diversity of intestinal flora. Dysbiosis of intestinal flora can lead to an imbalance in the ratio of primary bile acids to secondary bile acids, causing disorders in signal metabolic pathways, thereby promoting the occurrence of metabolic diseases such as obesity (Sayin et al., 2013). Rats with non-alcoholic fatty liver model fed a high-fat diet mainly showed increased in secondary bile acids (Jiao et al., 2018), and a significant imbalance in the ratio of primary and secondary bile acids. Arab et al. (2017) found that secondary bile acids are associated with the occurrence of non-alcoholic fatty liver disease. The mechanism may be that the synthesis of secondary bile acids leads to increasing permeability of the intestinal barrier, further inducing endotoxemia, resulting in insulin resistance, finally promoting the occurrence of metabolic diseases such as obesity.

A study has shown that BA also plays a role in regulating metabolism as hormones or signaling molecules (Houten et al., 2006). Bile salt hydrolase (BSH) in the intestine can dissociate taurine-and glycine-bound bile acids to form free bile acids. Bile acids converted by intestinal microorganisms can activate bile acid receptors in the host liver, intestine, and peripheral tissues, such as farnesoid X receptor (FXR) and G protein-coupled bile acid receptor 5 (TGR5), thereby activating different signaling pathways to regulate enterohepatic circulation and also regulate triglyceride, cholesterol, energy, and glucose homeostasis. The FXR-mediated signaling pathway is crucial for bile acid synthesis and metabolism. Primary bile acids can increase insulin sensitivity in mice, inhibit lipogenesis, and further promote fatty acid oxidation by activating FXR signaling, while secondary bile acids inhibit FXR signaling and thus affect energy metabolism (Kakiyama et al., 2013). Among them, TGR5 is widely expressed throughout the body, especially in brown adipose tissue. BA can promote lipolysis through TGR5 and play an important role in regulating energy metabolism. The occurrence of obesity is related to the browning of fat. A study found (Perino et al., 2014) that macrophage-specific TGR5 deficiency exacerbated insulin resistance in obese mice, while drug activation of TGR5 significantly reduced LPS induced macrophage chemokine expression. BA activates TGR5 to promote intracellular mitochondrial respiration to increase thermogenesis in mouse and human brown adipocytes (Broeders et al., 2015). Furthermore, TGR5 activation in white adipocytes participates in the browning process by increasing fatty acid *β*-oxidation and improving mitochondrial function (Velazquez-Villegas et al., 2018). In addition to acting on G protein-coupled receptors in the intestine, bile acid receptors are also present in adipose tissue. Studies have shown that bile acids can promote beigeization of adipose tissue, increase heat production, and improve energy metabolism (Broeders et al., 2015). Bile acids alter the composition of the intestinal microbiota and affect thermogenesis in white adipose tissue to improve glucose homeostasis and increase energy expenditure (Voreades et al., 2014).

In addition, bile acids can regulate appetite through the brain-gut axis. Studies have shown that bile acids bind to FXR receptors in the intestine to produce fibroblast growth factor 19/15 (FGF19/15), which reaches the hypothalamus through the brain-gut axis to regulate glucose metabolism, FGF19 in the hypothalamus has the effect of suppressing appetite and regulating energy metabolism (Kübeck et al., 2016; Zhi et al., 2019). Bile acids can also pass through the blood–brain barrier and bind to TGR5 and FXR to alleviate high-fat diet-induced obesity and improve energy metabolism (Santos-Marcos et al., 2019).

#### 2.3.4 Peptidoglycan (PGN)

PGN is a unique component of the cell wall in Gram-positive bacteria. Like TLR4, TLR2 can also induce innate immune responses by recognizing pathogen associated molecular patterns (PAMPs) and some endogenous toxic substances. The occurrence of obesity is often accompanied by inflammatory response, which is closely related to the activation of innate immunity. PGNs of different bacteria activate multiple signals through TLR2, such as NF-κB and JNK. When PGN is recognized by the innate immune system as PAPM, it activates a series of intracellular signaling pathways such as NF-κB and induces the synthesis of antimicrobial peptides (Pazos and Peters, 2019). Activation of the NF-κB signaling pathway leads to chronic inflammation and fat accumulation, thus promoting obesity.

Nucleotide-binding Oligomerization Domain containing 2 (NOD2) is a product of peptidoglycan that is located in epithelial and immune cells. This cytosol is critical for inflammatory immune responses and can affect mucosal bacterial colonization. NOD2 is a pattern recognition receptor that regulates the host innate immune response and reduces inflammation, inhibiting steatosis and obesity (Gurses et al., 2020). Increased expression of NOD2 has been reported in a range of human metabolic diseases, including obesity, diabetes, nonalcoholic fatty liver disease, and metabolic syndrome (Cavallari et al., 2020). Studies have shown that mice lacking NOD2 appeared hyperglycemia, adipose tissue and liver inflammation under high-fat diet conditions, demonstrating that the NOD2-regulated microbiota can alleviate diet-induced obesity and metabolic dysfunction in mice (Rodriguez-Nunez et al., 2017). PGN also plays an important role in insulin resistance and metabolic inflammation. Jin et al. (2020) found that PGN significantly increased the triglyceride content and the expression of lipogenesis-related genes in primary hepatocytes. In addition, peptidoglycan can inhibit the browning of adipose tissue (Chen H. et al., 2021). Studies have shown that PGN can inhibit white fat browning by activating TLR2-NF-κB signaling through adipocytes. At the same time, intestinal flora can produce peptidoglycan, which can stimulate inflammatory cells to trigger an immune response. In particular, when the permeability of the intestinal barrier changes, peptidoglycan is easily bound to the vascular endothelium and promotes the progression of atherosclerosis (Chistiakov et al., 2015). Therefore, intestinal microbiota regulates the occurrence of obesity by secreting peptidoglycan.

#### 2.3.5 Trimethylamine (TMA)

Choline is an important component of cell membranes. Choline is metabolized by microbial enzymes to produce TMA, which is further metabolized in the liver by flavin containing monooxygenase 3 (FMO3) to produce trimethylamine N-oxide (TMAO). Some scholars have demonstrated through experiments that there is a significant correlation between plasma TMAO levels and body mass index, which may be the result of affecting the intestinal microbes (Chen et al., 2024). Study had found that TMAO was involved in the energy metabolism of obese mice, meanwhile FMO3 was found in white adipose tissue, which was positively correlated with body mass index (Schugar et al., 2017). Targeted inhibition of the TMAO pathway by the gut microbiota protects mice from diet-induced obesity or leptin deficiency-related metabolic disorders (Schugar et al., 2022). Obesity is an important risk factor for atherosclerosis and coronary heart disease. Atherosclerosis, as the main pathophysiological basis of coronary heart disease, involves lipid metabolism and inflammatory response. Metabolomics technology has revealed that TMAO has pro-atherosclerotic properties and can be used as a potential biomarker for predicting the occurrence of cardiovascular diseases (Kong et al., 2024). Wang et al. (2011) first discovered in 2011 that TMAO plays a key role in the development of atherosclerosis; Ma and Li (2018) confirmed that TMAO can cause endothelial dysfunction. TMAO produced by intestinal flora enters the blood after being absorbed by intestinal epithelial cells, affecting the host’s metabolic capacity. These metabolites have been shown to affect obesity, insulin resistance, atherosclerosis and coronary heart disease (Schiattarella et al., 2019; Tang et al., 2019; Xu et al., 2020). Koeth et al. (2013) found that feeding mice with TMAO could lead to down-regulation of bile acid synthase expression, thereby inhibiting bile acid synthesis and causing fat deposition. At the same time, trimethylamine can also affect the expression of cholesterol 7α-hydroxylase and bile acid transporter. When the intestinal flora is unbalanced, resulting in excessive accumulation of TMA, a large amount of TMAO may be produced under the action of FMO3 in the liver, which can further cause metabolic disorders and obesity (Lin et al., 2012).

However, some studies have suggested that TMAO has no significant promoting effect on the occurrence of obesity. When studying the relationship between TMAO and aortic sclerosis, Collins et al. (2016) found that TMAO levels were inversely proportional to the degree of lesions in the aortic root and thoracic aorta. High levels of TMAO were significantly correlated with a reduction in the area of aortic lesions. At the same time, plasma lipid and lipoprotein levels were not correlated with TMAO. Due to the mouse model used in this experiment was ApoE (−/−) transgenic mice, which may be the reason affecting the experimental results. Gao et al. (2020) found that serum TMAO levels were positively correlated with age in the general population, while serum TMAO levels were not significantly correlated with obesity. In human studies, the association between serum TMAO and obesity has been inconsistent, which may be due to the different dietary patterns and genetic factors of the participants. Therefore, factors that may affect the experimental results need to be excluded in the study to ensure the reliability of the experimental results and to confirm the effect of TMAO on obesity. The relationships between gut microbial metabolites and obesity-related phenotypes summarized in this article are shown in Table 2.

**Table 2:** The relationships between gut microbial metabolites and obesity-related phenotypes.

Gut microbial metabolites	Phenotypic changes	References
Short-chain Fatty Acids, SCFAs	BWG↓, FBG↓, BW↓, IR↓, 5-HT↑, GIP↑, BFR↓, IL-6↓, IL-1*β*↓	Blaak et al. (2020), Den Besten et al. (2015), Larraufie et al. (2018), and Layden et al. (2012)
	ATP↑、GLP-1↑、PYY↑	
Lipopolysaccharide, LPS	IL-4↑, IL-8↑, IL-1↑, IL-6↑, TNF-*α*↑, BFR↑, BW↑, IR↑	Maruta et al. (2016) and Yamashita et al. (2009)
Bile Acid, BA	TC↓, TG↓, ATP↑, HDL-C↑, LDL-C↓, FBG↓	Santos-Marcos et al. (2019) and Jiao et al. (2018)
Peptidoglycan, PGN	IL-1↑, IL-6↑, IL-8↑, IL-12↑, TNF-*α*↑	Chen H. et al. (2021) and Gurses et al. (2020)
Trimethylamine, TMA	RCT↓, BMI↑, IR↓, BW↑, ATP↓	Schugar et al. (2017) and Gurses et al. (2020)

## 3 Conclusion and perspectives

**Figure 1:**
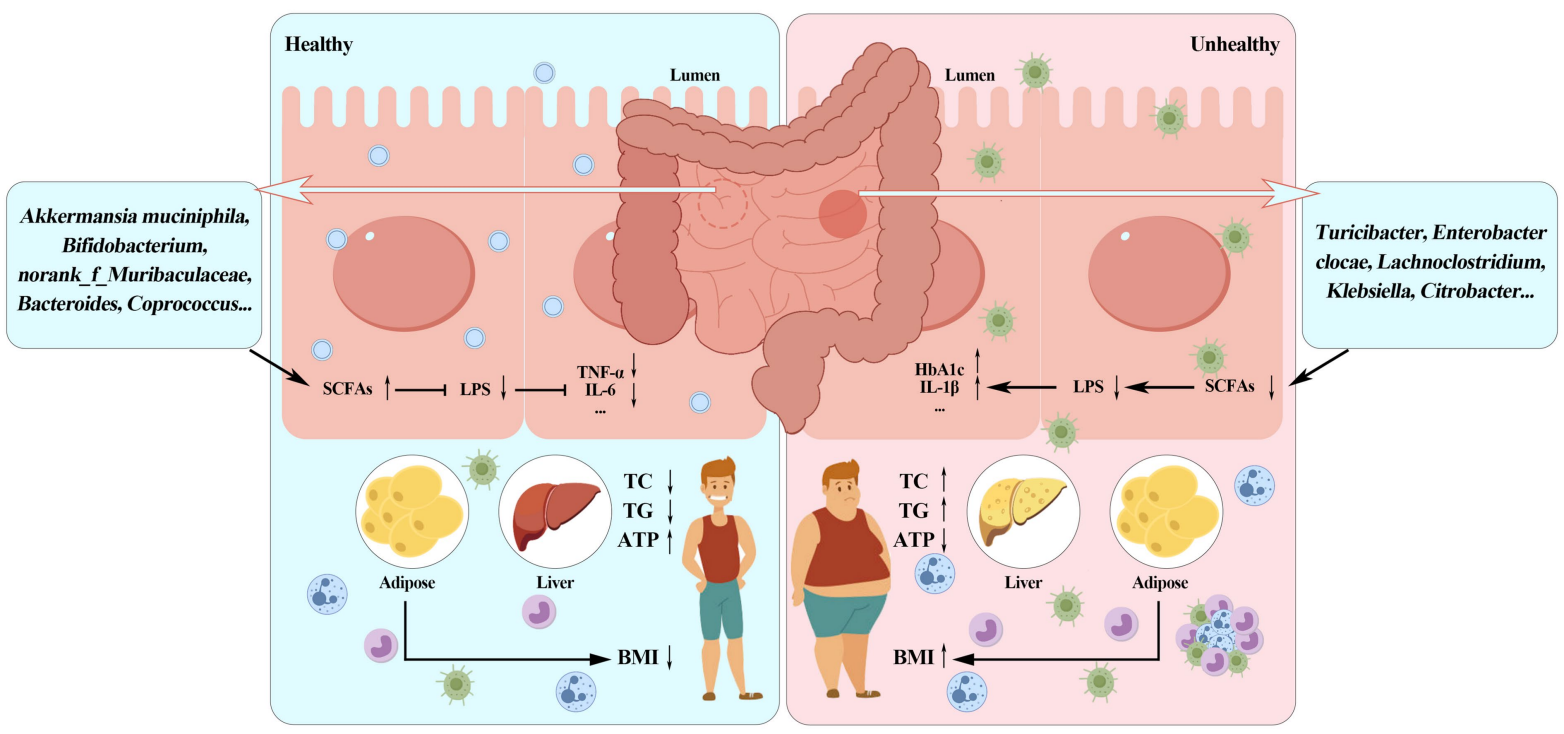
Schematic diagram of the relationship among gut microbiota, microbial metabolites and obesity.

Currently, the interaction between intestinal flora and body metabolism has become a hot topic. Intestinal microbiota is closely related to various metabolic diseases such as obesity. It fully participates in the digestion and absorption of nutrients and the metabolism of energy in the body. Changes in microbial diversity and metabolites affect the occurrence and development of obesity. As shown in Figure 1, beneficial bacteria such as *Bifidobacterium* and *Lactobacillus* can prevent the occurrence of obesity by strengthening the intestinal barrier, improving insulin sensitivity and metabolic disorders; Pathogenic bacteria such as *Escherichia coli*, *Ruminococcaceae*, and *Desulfovibrionaceae* are closely related to the formation of obesity; Metabolites produced by intestinal microbes, such as short-chain fatty acids, lipopolysaccharides and bile acids, play an important role in the prevention and treatment of obesity. With the further development of research, the association mechanism among intestinal flora, its metabolites and metabolic diseases such as obesity has been revealed, however, the intestinal microorganisms are a large and complex system. The interaction between the intestinal flora and the host, as well as the connection between the intestinal flora and various tissues and organs, still need to be explored, for example: studies in recent years have shown that adipose tissue is involved in immune responses. From an immunological perspective, what kind of dialogue takes place between intestinal flora and adipose tissue? On the other hand, with the continuous development of technology in recent years, in addition to metagenomics and 16S rDNA sequencing, the combined application of multi-omics such as single-cell transcriptomics, spatial transcriptomics and quantitative metabolomics will also help to explore the relationship between intestinal flora and obesity. This review highlights the pivotal role of gut microbiota and its metabolites in obesity pathophysiology. Future research should focus on translating microbiome-based findings into targeted clinical interventions, such as personalized probiotic therapies and dietary modifications, to combat obesity more effectively. In conclusion, intestinal microbes and its metabolites can be used as probiotics in clinical intervention in order to provide more solutions for the prevention and treatment of obesity in the future.
